# Detection of *Plasmodium vivax* and *Plasmodium falciparum* using colorimetric loop-mediated isothermal amplification from dried blood spots collected in active and passive malaria surveillance in the Peruvian Amazon

**DOI:** 10.1371/journal.pntd.0014366

**Published:** 2026-10-13

**Authors:** Oscar Nolasco, Joaquín Gómez, Hugo Rodríguez-Ferrucci, Dionicia Gamboa, Christopher Delgado-Ratto

**Affiliations:** 1 Laboratorio de Malaria: Parásitos y Vectores, Laboratorios de Investigación y Desarrollo, Facultad de Ciencias e Ingeniería, Universidad Peruana Cayetano Heredia, Lima, Peru; 2 Comunidad de conocimiento GIBBS-UNFV, Facultad de Ciencias Naturales y Matemática, Universidad Nacional Federico Villarreal, Lima, Peru; 3 Malaria Research Group (MaRch), Global Health Institute, Family Medicine and Population Health Department, Faculty of Medicine and Health Sciences, University of Antwerp, Antwerp, Belgium; 4 Facultad de Medicina Humana, Universidad Nacional de la Amazonía Peruana (UNAP), Iquitos, Loreto, Peru; 5 Departamento de Ciencias Celulares y Moleculares, Facultad de Ciencias e Ingeniería, Universidad Peruana Cayetano Heredia, Lima, Peru; Advanced Centre for Chronic and Rare Diseases, INDIA

## Abstract

**Background:**

As malaria transmission declines in regions approaching elimination, highly sensitive diagnostic tools are needed to detect low-density infections, particularly in active and passive surveillance settings. Loop-mediated isothermal amplification (LAMP) is a promising molecular alternative to conventional diagnostics. This study evaluated the performance of a colorimetric LAMP (cLAMP) assay for detecting *Plasmodium vivax* and *Plasmodium falciparum* from dried blood spots (DBS) in the Peruvian Amazon.

**Methodology/Principal findings:**

The cLAMP assay was applied to DNA extracted from DBS collected through active case detection (ACD; n = 574) and passive case detection (PCD; n = 431). Two extraction methods were compared: mini spin column (MSC DNA) and a boil-and-spin (BS DNA) method. Diagnostic performance was evaluated against microscopy and real-time quantitative PCR (qPCR).

In ACD samples, qPCR identified 125 malaria-positive cases (117 *P. vivax*, 8 *P. falciparum*). Microscopy showed a sensitivity of 57.6%, compared with 63.2% for cLAMP using BS DNA and 82.4% using MSC DNA. In PCD samples, qPCR detected 140 positives (129 *P. vivax*, 9 *P. falciparum,* and 2 mixed infections). Microscopy sensitivity was 72.9%, while cLAMP reached 77.1% with BS DNA and 82.1% with MSC DNA. Specificity remained high (> 99.0%) across methods.

**Conclusions/Significance:**

cLAMP improved the detection of malaria infections from DBS compared with microscopy when combined with column-based DNA extraction in active surveillance activities. While the boil-and-spin method showed performance comparable to microscopy, column-based extraction substantially increased sensitivity, especially for low-density infections identified through active surveillance. These findings support cLAMP as a practical and sensitive complementary tool for malaria surveillance in low-transmission and elimination settings.

## Author summary

*Plasmodium vivax* and *Plasmodium falciparum* are the main causes of malaria in the Peruvian Amazon. Diagnosis in these areas relies mainly on microscopy and rapid tests, which are widely used but can miss infections when parasite levels are low. This is especially important in remote communities, where access to well-equipped laboratories and highly trained staff is limited. As countries move toward eliminating malaria, it becomes essential to detect even low-level and asymptomatic infections that can sustain transmission. This requires more sensitive tools that can be used in hard-to-reach settings. Loop-mediated isothermal amplification (LAMP) is a molecular method that can detect infections at lower levels than routine tests while remaining relatively simple to use. In this study, we evaluated a colorimetric LAMP (cLAMP) test using dried blood spots collected in the Peruvian Amazon and analysed in a laboratory in Lima. We found that cLAMP detected more infections than microscopy, particularly those with low parasite levels. Because dried blood spots are easy to collect, store, and transport, this approach could help improve malaria surveillance and support national efforts to eliminate the disease, especially in remote endemic regions.

## Introduction

Despite a substantial global reduction in malaria incidence and mortality, morbidity remains high in many tropical and subtropical regions [1]. The burden of malaria in Peru is concentrated in the Amazon basin. In 2025, 99.0% (33186/33526) of reported cases were from the Loreto region, where *P. falciparum* constituted 15.6% (5175/33169) and *P. vivax* 84.4% (27984/33169) of cases [2]. Malaria predominantly affects the peri-urban, rural, and remote Amazonian population, where access to healthcare is limited and often requires travel of one to five days to reach primary point of care (PoC) with the basic laboratory infrastructure [3].

In addition to geographical and logistical limitations that limit health care access in hard-to-reach areas, Peruvian malaria elimination efforts are challenged by the high prevalence of asymptomatic and submicroscopic infections. As routine surveillance relies on PCD, microscopy, and RDTs, asymptomatic and submicroscopic infections remain undetected and contribute to the persistence of malaria transmission [4]. In this context, the Peruvian Ministry of Health (MoH) implemented the Plan Malaria Cero (PMC), an intensified malaria control strategy with a pre-elimination orientation, in 2017, which focused on active case detection, prompt treatment, and vector control using insecticide-treated mosquito nets in endemic areas [5]. Although the PMC achieved a substantial reduction in malaria cases, with a 56.0% decrease between 2017 and 2019, access to malaria services was disrupted during the COVID-19 pandemic, coinciding with an apparent 63.8% reduction in reported cases between 2019 and 2020, which may reflect underreporting. To consolidate previous gains, the MoH subsequently launched the National Malaria Elimination Plan 2022–2030 (NMEP), which emphasizes implementing new active surveillance strategies and aims to reduce malaria cases by 90% by 2030 [6].

Currently, malaria case detection in Peru is performed through routine microscopy and rapid diagnostic tests (RDTs) [7,8]. However, misdiagnosis by microscopy often occurs at levels below 100 parasites/µL due to laboratory infrastructure and inadequately skilled healthcare workers [9–11]. Likewise, RDTs have lower sensitivity, with a limit of detection of 100–200 parasites/μL [9]. In addition, HRP2-only tests in Peru are no longer included in the MoH strategies due to the widespread *pfhrp2* deletion in the Peruvian *P. falciparum* population, extremely low sensitivity (non-*P. falciparum: 77%*; *P. falciparum:* 54%*),* and cross-reaction with *P. vivax* [12,13].

These limitations highlight the need to strengthen the implementation of sensitive diagnostic tools suited for point-of-care use. LAMP has been included in the most recent update of the Peruvian technical guideline for malaria diagnosis; however, its implementation in remote settings remains limited. LAMP represents a promising molecular approach that enables DNA amplification under isothermal conditions and allows visual detection of amplification products without sophisticated equipment [14–16]. The versatility of LAMP has facilitated its application in ACD and PCD settings, where it has successfully detected low-density *Plasmodium falciparum* and *P. vivax* infections among asymptomatic individuals in community-based studies in Cambodia and Thailand, revealing hidden reservoirs of infection that were missed by microscopy and RDTs, thereby enabling targeted treatment interventions [17,18]. In Peru, both commercial and in-house LAMP assays have demonstrated high diagnostic performance with whole-blood samples [19–22]. In a previous study, we reported a cLAMP assay targeting multicopy genomic regions, *cox1* for *P. vivax* and *Pfr364* for *P. falciparum*, combined with the neutral red dye, which showed good sensitivity for the detection of submicroscopic infections from whole blood samples [23].

In the present study, we evaluated the diagnostic accuracy of this cLAMP assay for detecting *P. vivax* and *P. falciparum* from DBS collected via ACD and PCD in remote endemic communities.

## Materials and methods

### Ethical statement

Ethical approval was obtained from the corresponding Ethics Review board from Universidad Peruana Cayetano Heredia (SIDISI Project: 201296 and SIDISI Project: 102725).

### Study area and sample collection

The study samples were collected in the remote community of Nueva Jerusalén in the basin of the Macusari River, a tributary of the Corrientes River, in the district of Trompeteros, Loreto region, Peru (Fig 1). The main access route requires approximately four hours of travel from the Andoas district (Province of Datem del Marañón) via a mountain trail. The local PoC is classified by the Peruvian MoH as a level I-1 facility and is staffed by a laboratory technician, an obstetrician, and a physician, serving approximately 452 inhabitants.

**Fig 1:**
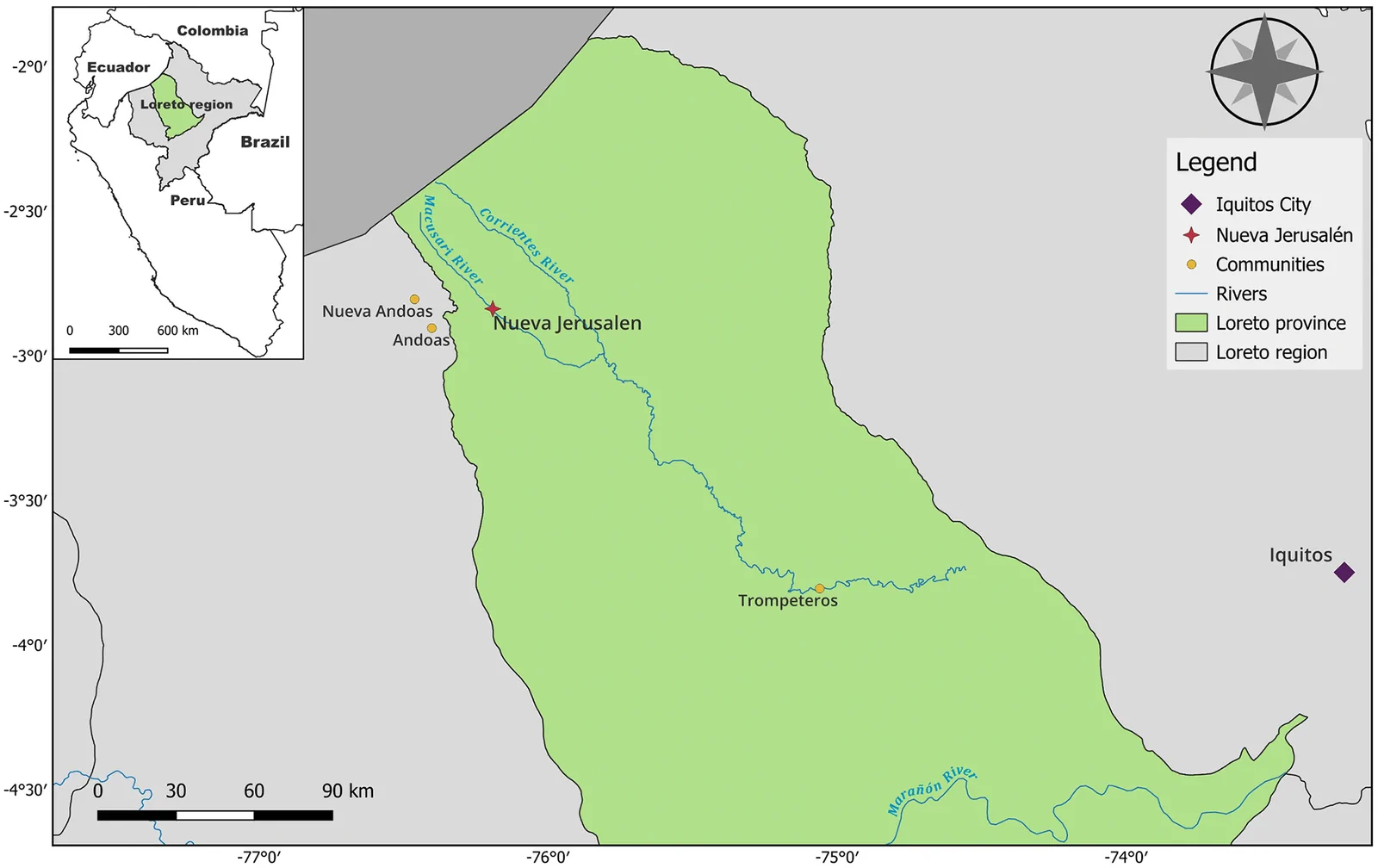
Map of the study area. In the left upper corner, an inset map highlights Peru’s location in South America, the Loreto region in gray, and the Loreto province in green. The main map shows the rivers in blue, neighboring communities in yellow, and the study community in red. The map was produced using QGIS 3.40 (QGIS development team 2025, QGIS Geographic Information System. Open-Source Geospatial Foundation Project. http://www.qgis.org/). Geographic datasets for country-level boundaries were obtained from Natural Earth (https://www.naturalearthdata.com/downloads/10m-cultural-vectors/10m-admin-0-countries). Administrative boundaries and river shapes within Peru were obtained from the National Water Authority (https://www.gob.pe/ana) and the National Institute of Statistics and Informatics (https://www.gob.pe/inei/). Peruvian geospatial datasets are freely available through the GEOIDP-GEOPERU platform for research. Terms of use can be found at: https://www.geoidep.gob.pe/static/docs/terminos_condiciones.pdf.

The community features wooden housing and a tropical climate with consistently high temperatures and humidity year-round. MoH reports indicate that malaria transmission is sustained year-round, with irregular seasonal peaks. Because these activities were conducted by the MoH, informed consent was not obtained from participants.

A total of 1084 DBS were collected on filter paper from November 6th to December 13th, 2019, through routine ACD and PCD conducted by local health personnel in collaboration with the MoH PMC. For PCD, samples were obtained from individuals with fever (> 37.5 °C) or suspected malaria symptoms at the health center. ACD samples were collected during three community-wide screening campaigns (Campaign dates: Nov. 16th-17th, Nov. 23rd-24th, Nov. 30th- Dec. 2nd) open to all residents, regardless of age, sex, or clinical status [24]. Microscopic diagnosis and subsequent treatment of malaria cases were performed by local health workers.

The DBS were transported to the UPCH Malaria laboratory in Lima, Peru, for molecular analysis. Upon receipt, samples were assessed for suitability for downstream testing, and only those with sufficient blood volume on the filter paper to perform both colorimetric LAMP (cLAMP) and real-time quantitative PCR (qPCR) were included. Samples with insufficient blood volume were excluded from further analysis. Finally, cLAMP and qPCR results were compared with those from routine microscopy. A schematic overview of the experimental workflow is shown in Fig 2, and detailed sample information is provided in the S1 File.

**Fig 2:**
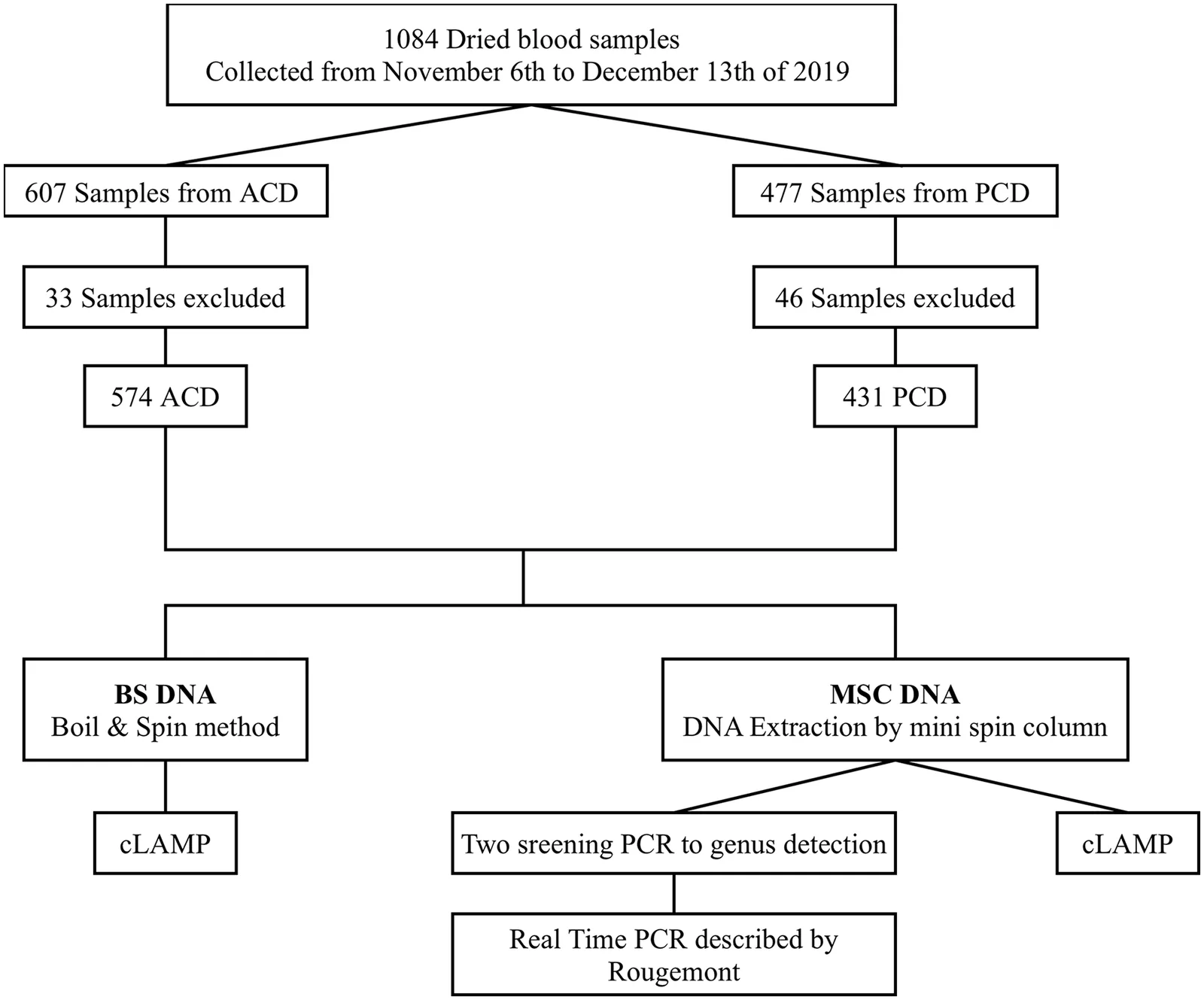
Flowchart of the experimental procedure. ACD: Active Case Detection; PCD: Passive Case Detection; BS DNA: Boil & Spin extracted DNA; MSC DNA: Mini spin column DNA.

#### PCD samples.

The PCD group includes 477 DBS on filter paper collected at the health center in Nueva Jerusalén on consecutive days from November 6th to December 13th of 2019 from febrile patients. Forty-six samples were excluded due to insufficient blood dots on the paper filter to perform cLAMP, resulting in a final set of 431 DBS samples included in the analysis. The passive surveillance dataset included no sociodemographic information.

#### ACD samples.

The ACD group includes 607 DBS on filter paper from participants in three population survey rounds, with a one-week interval between active sample collections: 16–17 of November; 23–24 of November; and 30 November to 1 December. Thirty-three samples were excluded because they had insufficient blood dots on the filter paper to perform cLAMP, leaving 574 DBS on filter paper. The active surveillance dataset provided by the MoH included no sociodemographic information.

### Ethical considerations

The use of previously collected samples for LAMP diagnosis in this study was approved by the Ethics Review Board of the Universidad Peruana Cayetano Heredia (SIDISI: 201296). The dried blood spot (DBS) samples analyzed were obtained from the UPCH Malaria Project (SIDISI 102725), which had been approved by the Ethics Review Board of UPCH and the Regional Health Directorate of Loreto (GERESA Loreto; Oficio N° 289–2018-GRL-DRS1-30.09-Investigación).

The DBS samples were originally collected by GERESA Loreto personnel as part of routine Ministry of Health malaria surveillance, diagnosis, and treatment activities rather than specifically for the present study. Therefore, individual written or verbal informed consent was not obtained at the time of sample collection. This also applied to samples collected from children; consequently, written or verbal consent from their parents or legal guardians was not obtained.

### Reference samples

Positive DNA control for *P. falciparum* was extracted from the 1st WHO International Standard for *P. falciparum* DNA (NIBSC code: 04/176) [25]. Positive DNA controls for *P. vivax* were extracted from a blood sample confirmed by microscopy and PCR. Control DNA samples were prepared at a dilution equivalent to 20 parasites/µL in a blood sample and included in each assay batch.

### Microscopy data

All samples have a semiquantitative report of parasite density according to the Peruvian MoH [8]. Parasite densities were recorded as the number of parasites detected per 100 microscopic fields when fewer than 40 parasites were observed. For higher parasite densities, the following scale was applied: “+/2” (40–60 parasites in 100 microscope fields), “+” (1 parasite per field in 100 fields), “++” (2–20 parasites per field in 100 fields), “+++” (21–200 parasites per field in 100 fields), and “++++” (>200 parasites per field in 100 fields).

### DNA extraction using Mini spin column (E.Z.N.A. Blood DNA Mini Kit)

The DNA was extracted using EZNA Blood DNA (Omega Bio-tek, Norcross, GA, USA) according to the manufacturer’s protocol. In brief, two discs of 6 mm diameter punched from the DBS sample on filter paper were transferred to a sterile microcentrifuge tube, adding 250 µL of 2X TEN buffer pH 8.0 (0.2 M NaCl, 0.02 M Tris-HCl pH 8.0, 0.002 M EDTA pH 8.0), 180 µL of distilled water, 50 µL of 10% SDS, and 25 µL of OB protease solution. The mix was incubated at 65°C for 60 minutes, centrifuged at 13 000 g for 5 min, and the supernatant was transferred to a new tube, to which 250 µL of BL buffer and 260 µL of 100% ethanol were added. 500 µL of this homogenate was transferred to a mini HiBind DNA column inserted in a 2 mL collecting tube and centrifuged at 10 000 g for 1 minute. The mini column was inserted into a new 2 mL collection tube, 500 µL of HBC buffer was added, and then the mixture was centrifuged at 10 000 g for 1 minute. 700 µL of wash buffer was added to the mini column, and the column was centrifuged at 10 000 g for 1 minute; this process was repeated once more. The empty HiBind mini-DNA column was centrifuged at maximum speed for 2 minutes to dry the column matrix. The DNA from the HiBind DNA mini column was eluted in 60 µL of the elution buffer preheated to 65°C. Eluted DNA was immediately used or stored at -20°C. Hereafter, the DNA extracted using this method is referred to as MSC DNA. Although no internal extraction controls were used, the EZNA Blood DNA extraction kit has been widely used in other works and reports consistent DNA yields from blood samples.

### Sample preparation using the boiling & spin extraction method

The Boil & Spin method was performed using two 6 mm diameter discs punched from the DBS sample on filter paper, with 80 µL of 1X lysis buffer (8 mM Tris, 80 mM NaCl, 0.08% SDS, pH 8.5) [22]. The mixture was homogenized and incubated in a water bath at 95 °C for 7 minutes. After that, the homogenate was centrifuged at 13 000 g for 7 minutes. Finally, the supernatant was recovered and used for the LAMP analysis. Hereafter, the DNA extracted using this method is referred to as BS DNA.

### Colorimetric LAMP (cLAMP) conditions

We performed a slight modification to the previously reported Pfr364-LAMP to detect *P. falciparum* and PvCOX1-LAMP to detect *P. vivax* [23]. The primer sequences were identical to those originally reported (S3 File), whereas modifications were introduced to the reaction volumes and assay conditions: The final volume of each reaction was 15 μL, containing 0.5 × Bst DNA polymerase reaction buffer (10 mM Tris-HCl, 5 mM (NH_4_)_2_SO_4_, 25 mM KCl, 1 mM MgSO_4_, 0.05% Tween 20, pH 8.8 New England Biolabs, MA), 0.2 μM of each external primer F3 and B3, 2 μM of each inner primer FIP and BIP, 12 U Bstv2.0 DNA polymerase (New England Biolabs Inc., MA), 1.4 mM of each dNTPs, 0.003% of neutral red dye and 5 μL of a test sample. The reaction mixture for PvCOX1-LAMP was adjusted to 6 mM of MgSO_4_, and 0.4 M betaine, and the Pfr364-LAMP was adjusted to 8 mM of MgSO4.

To ensure the quality of the process, each group of 93 samples was evaluated together with two positive controls (one for *P. vivax* and one for *P. falciparum,* each containing 20 parasites/μL) and a negative control without DNA.

The LAMP was performed in PCR reaction tube strips. A positive reaction was assessed by visual inspection and considered as such only if the reaction turned from yellow to fuchsia after 65 min of incubation at 60°C. Incubation steps were performed using a Bio-Rad T100 Thermal Cycler (Bio-Rad Laboratories, CA, USA). Notably, the assessment of cLAMP results was blinded to microscopy and qPCR to reduce the potential for observer bias.

### Species-specific detection by real-time qPCR

To maximize malaria parasite detection and minimize the risk of false-negative results, all samples were initially screened with two independent genus-specific real-time PCR assays targeting distinct genomic regions: the *18S rRNA* and the mitochondrial *COXIII* gene. Samples that amplified in at least one screening assay were then evaluated with a species-specific TaqMan qPCR to identify *P. falciparum* and *P. vivax*.

The first screening targeting the *18S rRNA* gene of human malaria parasites was described by Pincelli *et al.* [26]. The reaction mixtures were prepared at a final volume of 25 μL, containing 5 μL of MSC DNA sample, with a final concentration of 1X PerfeCTa SYBR Green FastMix (Quantabio, Beverly, Massachusetts), and 0.2 μM of each oligonucleotide primer (Plasmo 1, 5′-GTT AAG GGA GTG AAG ACG ATC AGA-3′ and Plasmo 2, 5′-AAC CCA AAG ACT TTG ATT TCT CAT AA-3′). The amplification was carried out on a CFX96 Touch Real-Time PCR Detection System (BioRad), for DNA amplification, with a template denaturation step at 95°C for 10 minutes (min), followed by 40 cycles of 15 seconds (sec) at 95°C, and 1 min at 60°C, with fluorescence acquisition at the end of each extension step. Amplification was immediately followed by a melting program consisting of 3 min at 70°C, and a stepwise temperature increase of 0.5°C/s until 90°C, with fluorescence acquisition at each temperature transition.

The second screening targets the 3’UTR mitochondrial *COXIII* gene using an *in-house* genus-specific PCR protocol. The reaction mixtures were prepared at a final volume of 25 μL, containing 5 μL of sample, with a final concentration of 1X PerfeCTa SYBR Green FastMix (Quantabio, Beverly, Massachusetts), and 0.2 μM of each oligonucleotide primer (COXIIIF, 5′- CGGTAGATAGGGAACAAACT -3′ and COXIIIR, 5′- CTTTGCCTGGAGGTTACG -3′). The amplification was carried out on a CFX96 Touch Real-Time PCR Detection System (BioRad), with a template denaturation step at 95°C for 3 min, followed by 45 cycles of 15 s at 95°C, 15 s at 60°C, and 15 s at 68°C, with fluorescence acquisition at the end of each extension step. Amplification was immediately followed by a melting program consisting of 3 min at 70 °C, then a stepwise temperature increase of 0.5 °C/s to 90 °C, with fluorescence acquisition at each temperature transition.

All samples that showed amplification with at least one screening test were evaluated using species-specific real-time TaqMan qPCR, as described by Rougemont *et al.* [27]. The reaction mixtures were prepared in a final volume of 20 µL, containing 5 μL of sample DNA with a final concentration of 1X of master mix TaqMan Universal Master Mix 2.0 (Applied Biosystems, Thermo Fisher Scientific, Waltham, MA, USA), 0.1 μM of each genus-specific unlabeled oligonucleotide primer, 0.08 μM of each species-specific labeled probe (*P. vivax*: 5′ VIC [Applied Biosystems proprietary green fluorescent dye]-AGC AAT CTA AGA ATA AAC TCC GAA GAG AAA ATT CT-QSY [Applied Biosystems proprietary quencher] 3′; *P. falciparum*: 5′ FAM [6-carboxyfluorescein]-AGC AAT CTA AAA GTC ACC TCG AAA GAT GAC T-QSY [Applied Biosystems proprietary quencher] 3′). Polymerase chain reaction amplification comprised an initial denaturation step at 95°C for 10 min, followed by 45 cycles of 15 s at 95°C and 1 min at 60°C. No-template controls (containing all reagents for amplification except for the DNA template) and positive controls (containing DNA for *P. falciparum* and *P. vivax* previously quantified) were run for every PCR microplate.

All the samples with a Cq (quantification cycle) value less than 36.4 were considered positive according to the Limit of Quantification (LoQ) determined using a serial dilution of the 1st WHO International Standard for *Plasmodium falciparum* DNA Nucleic Acid Amplification Techniques available at the Malaria laboratory-UPCH. Because a quantified *P. vivax* reference standard was not available, we did not determine a LoQ for this species and used the same threshold as for *P. falciparum*. Samples with Cq values between 36.4 and 38.5 were retested using a threefold increase in DNA input, and positivity was confirmed by repeat amplification.

### Statistical analysis

Laboratory data were coded and entered into Microsoft Excel. Diagnostic performance indicators, including sensitivity, specificity, and area under the curve (AUC), were calculated using the pROC (version 1.19) package in R (version 4.5.0; R core team 2025) implemented in RStudio (version 2026.01.2 + 418) and compared using DeLong’s test. Holm-Bonferroni corrections for multiple testing were applied when necessary. Quantitative variables were summarized using means or medians, as appropriate.

Agreement between diagnostic methods was assessed using Cohen’s kappa coefficient and the McNemar test. Kappa values were interpreted as follows: κ < 0.20 (poor agreement), 0.21–0.40 (fair), 0.41–0.60 (moderate), 0.61–0.80 (good), 0.81–0.99 (very good), and 1.00 (perfect agreement). Differences in parasitemia levels between diagnostic methods and surveillance strategies were evaluated using the Wilcoxon rank-sum test with Bonferroni correction.

## Results

### Malaria parasite detection in PCD samples

A total of 431 PCD samples were included in this study. Microscopy identified 102 positives (97 *P. vivax* and 5 *P. falciparum*). Real-time qPCR detected 140 positives, comprising 129 *P. vivax*, 9 *P. falciparum,* and 2 mixed infections. cLAMP (Pfr364-LAMP and PvCOX1-LAMP) using BS DNA detected 109 positives (100 *P. vivax*, 7 *P. falciparum,* and 2 mixed infections), whereas cLAMP using MSC DNA detected 116 positives (109 *P. vivax*, 5 *P. falciparum,* and 2 mixed) (Table 1).

**Table 1:** Malaria prevalence of qPCR, microscopy, and cLAMP methods, and diagnostic accuracy using qPCR as the reference method in PCD samples (N = 431).

Species	Real-time qPCR	Microscopy	cLAMP – BS DNA	cLAMP – MSC DNA
*P. vivax*	129	97	100	109
*P. falciparum*	9	5	7	5
Mixed	2	0	2	2
Negative or non-P. *vivax* or *P. falciparum*	291	329	322	315
Malaria Sensitivity [95% CI]		72.90% [65-80.2]	77.10% [70.2-84.1]	82.10% [75.8- 88.5]
Malaria Specificity [95% CI]		100% [100-100]	99.70% [99-100]	99.70% [99-100]
AUC [95% CI]		0.86 [0.82-0.90]	0.88 [0.85-0.92]	0.91 [0.87-0.94]
Agreement Cohen’s Kappa [95% CI]		0.78 [0.72-0.85]	0.81 [0.76-0.87]	0.86 [0.80-0.91]

### Malaria parasite detection in ACD samples

A total of 574 samples from ACD were analyzed. Microscopy identified 72 positives (66 *P. vivax*, 6 *P. falciparum*); real-time qPCR detected 125 positives (117 *P. vivax,* 8 *P. falciparum*); cLAMP using BS DNA detected 79 positives (72 *P. vivax*, 6 *P. falciparum,* and 1 mixed infection); and cLAMP using MSC DNA detected 104 positives (97 *P. vivax,* 7 *P. falciparum*) (Table 2).

**Table 2:** Malaria prevalence of qPCR, microscopy, and cLAMP methods, and diagnostic accuracy using qPCR as the reference method in ACD samples (N = 574).

Species	Real-time qPCR	Microscopy	cLAMP – BS DNA	cLAMP – MSC DNA
*P. vivax*	117	66	72	97
*P. falciparum*	8	6	6	7
Mixed	0	0	1	0
Negative or Non-*P. vivax* or *P. falciparum*	449	502	495	470
Malaria Sensitivity [95% CI]		57.60% [48.9-66.3]	63.20% [54.7-71.7]	82.40% [75.7- 89.1]
Malaria Specificity [95% CI]		100.00% [100-100]	100.00% [100-100]	99.80% [99.3-100]
AUC [95% CI]		0.79 [0.75-0.83]	0.82 [0.77-0.86]	0.91 [0.88-0.95]
Agreement Cohen’s Kappa [95% CI]		0.68 [0.60- 0.76]	0.73 [0.66 - 0.80]	0.87 [0.83- 0.92]

### Diagnostic accuracy

Using qPCR as the reference method, microscopy in PCD samples showed a sensitivity of 72.9% (95% CI [65-80.2]) and specificity of 100%. The sensitivity of cLAMP was 77.1% (95% CI [70.2-84.1]) for BS DNA and 82.1% (95% CI [75.8- 88.5]) for MSC DNA, while specificity remained high (99.7%, 95% CI [99–100]) for both methods (Table 1). Diagnostic accuracy increased accordingly, with AUC values of 0.86 for microscopy, 0.88 for cLAMP–BS DNA, and 0.91 for cLAMP–MSC DNA. Agreement with qPCR also improved, with Cohen’s kappa increasing from 0.78 for microscopy to 0.86 for cLAMP using MSC DNA (Table 1).

In ACD samples, microscopy showed low sensitivity (57.6%) despite perfect specificity, whereas cLAMP increased sensitivity to 63.2% with BS DNA and to 82.4% with MSC DNA, with specificity ≥ 99.8% for both extraction methods. Overall diagnostic performance was highest for cLAMP using MSC DNA, as reflected by the highest AUC (0.91) and strongest agreement with qPCR (k = 0.87) (Table 2).

Comparative analysis of AUCs using DeLong’s test with a Holm correction for multiple comparisons found no significant differences in microscopy and cLAMP performance between PCD groups, regardless of the DNA extraction method. In contrast, in ACD samples, cLAMP using MSC DNA outperformed microscopy (p < 0.001), whereas cLAMP using BS DNA performed comparably (p = 0.125) (Fig 3).

**Fig 3:**
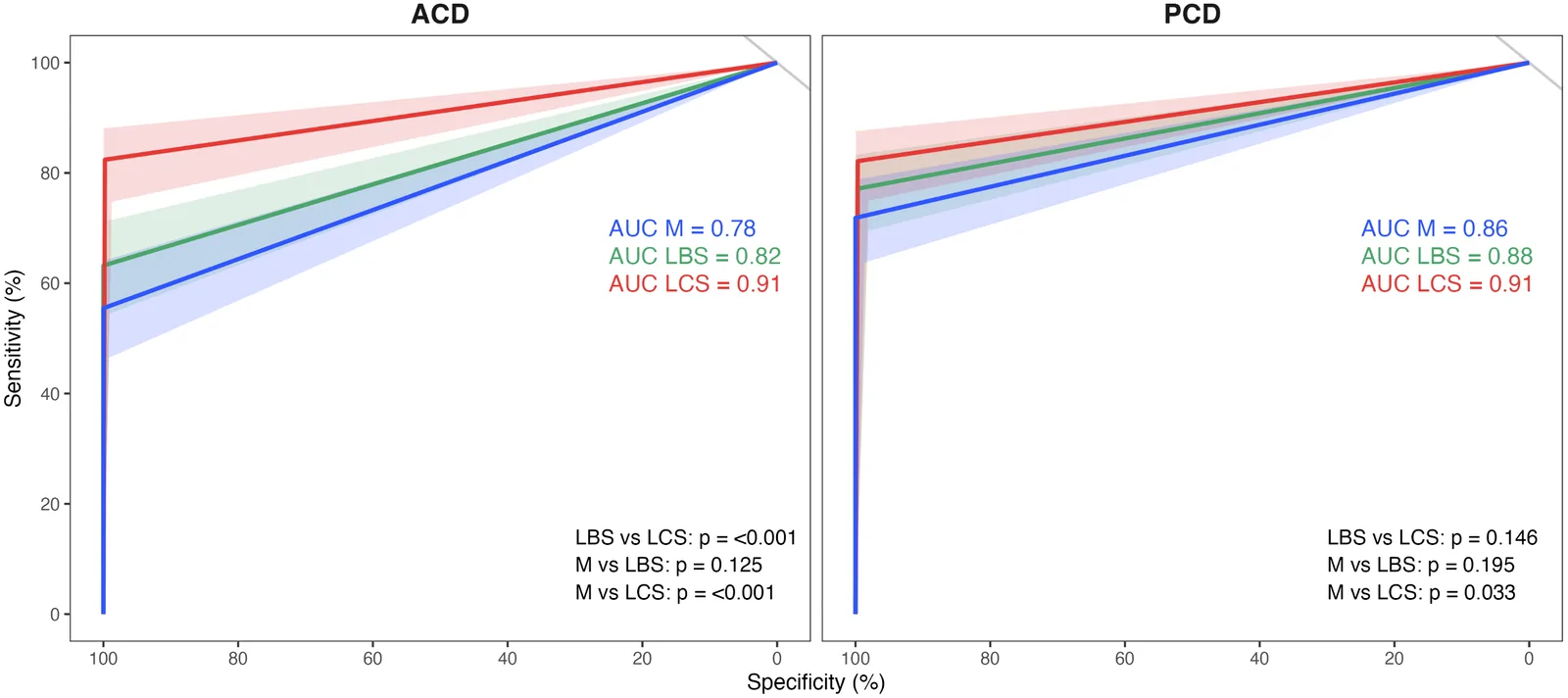
Comparative AUC for diagnostic methods in ACD and PCD. Each solid line represents the AUC estimate. Surrounding lighter colors represent the 95% CI. Abbreviations: M, microscopy; LBS, cLAMP using BS DNA; LCS, cLAMP using MSC DNA.

A small number of discordant results were observed across diagnostic methods for PCD samples. In PCD samples, two samples were positive by cLAMP but negative by qPCR: one using BS DNA (code 10369) and one using MSC DNA (code 10610). Moreover, four samples with qPCR-determined parasite densities below 10 parasites/µL were negative by both cLAMP methods. Additionally, two samples that were positive by microscopy and qPCR were negative by cLAMP using BS DNA (one with a parasite density of <10 parasites/µL and the other 85 parasites/µL by PCR), and two samples were negative by cLAMP using MSC DNA. All discordant samples showed low parasite densities as quantified by qPCR (<10 parasites/µL) (Fig 4).

**Fig 4:**
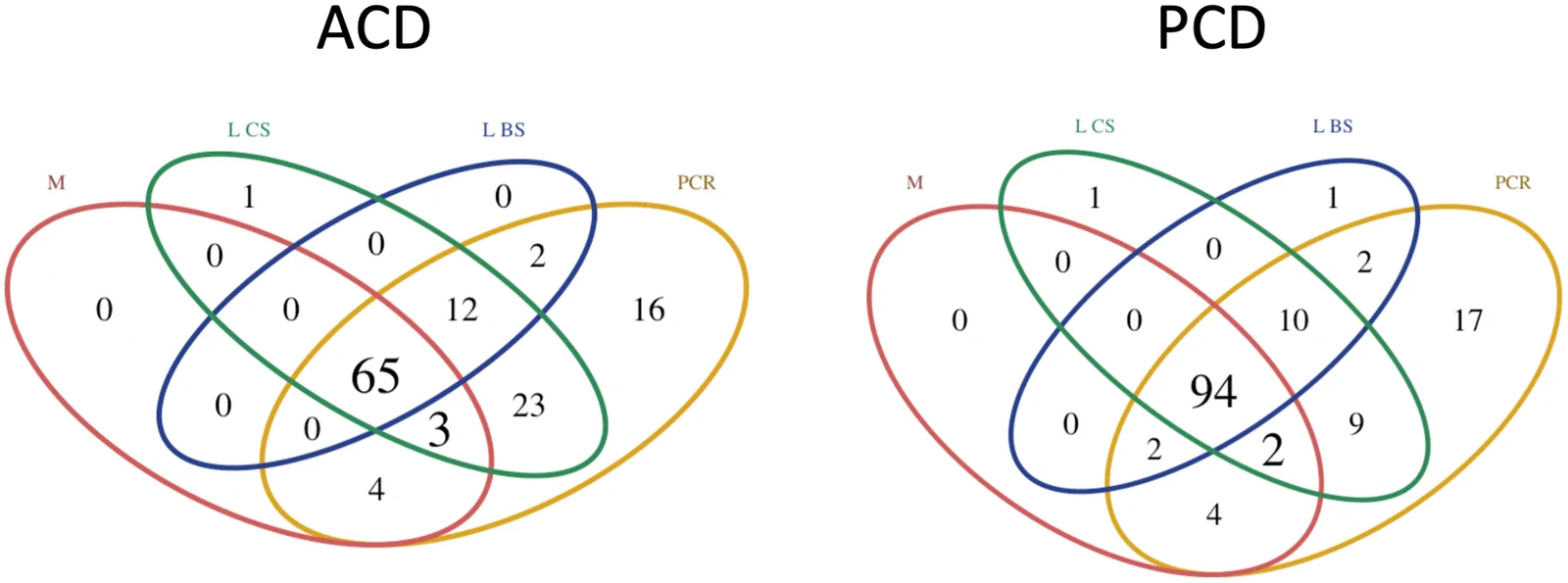
Venn diagram showing the distribution of samples across diagnostic tests. The diagram displays positive samples detected by microscopy **(M)**, cLAMP using BS DNA (LBS), cLAMP using MSC DNA (LCS), and qPCR, grouped by active case detection (ACD; N = 574) and passive case detection (PCD; N = 431). M, microscopy; LBS, cLAMP using BS DNA; LCS, cLAMP using MSC DNA; PCR, polymerase chain reaction.

In ACD samples, one additional *P. vivax* infection (code 10230) was detected exclusively by cLAMP using MSC DNA. Four samples positive by microscopy and qPCR were negative by both cLAMP methods, and three additional samples were negative only by cLAMP using BS DNA. All discordant samples exhibited low parasite densities as measured by qPCR (<70 parasites/µL by PCR or <6 parasites per 100 microscopy fields by microscopy) (Fig 4).

### Effect of malaria parasite density by diagnostic test

Parasite density varied significantly between active and passive surveillance strategies. Samples from passive surveillance generally had higher parasite loads, whereas active surveillance identified a larger proportion of low-density infections. A significant difference in qPCR parasite densities was observed between active and passive surveillance (Wilcoxon rank-sum test, p = 0.00025).

In ACD samples, 66.4% (83/125) of qPCR-positive samples had parasite densities below 100 parasites/µL, compared with 39.3% (55/140) in PCD. Microscopy detected 37.3% (31/83) of low-density infections in ACD samples, whereas cLAMP detected 44.6% (37/83) with BS DNA and 73.5% (61/83) with MSC DNA. In PCD, microscopy detected 32.7% (18/55) of samples with parasite densities below 100 parasites/µL, while cLAMP detected 41.8% (23/55) with BS DNA and 54.5% (30/55) with MSC DNA.

Both cLAMP approaches reliably detected infections with parasite densities above 100 parasites/µL. However, cLAMP using MSC DNA consistently showed superior sensitivity for low-density infections compared with microscopy and cLAMP using BS DNA (Fig 5).

**Fig 5:**
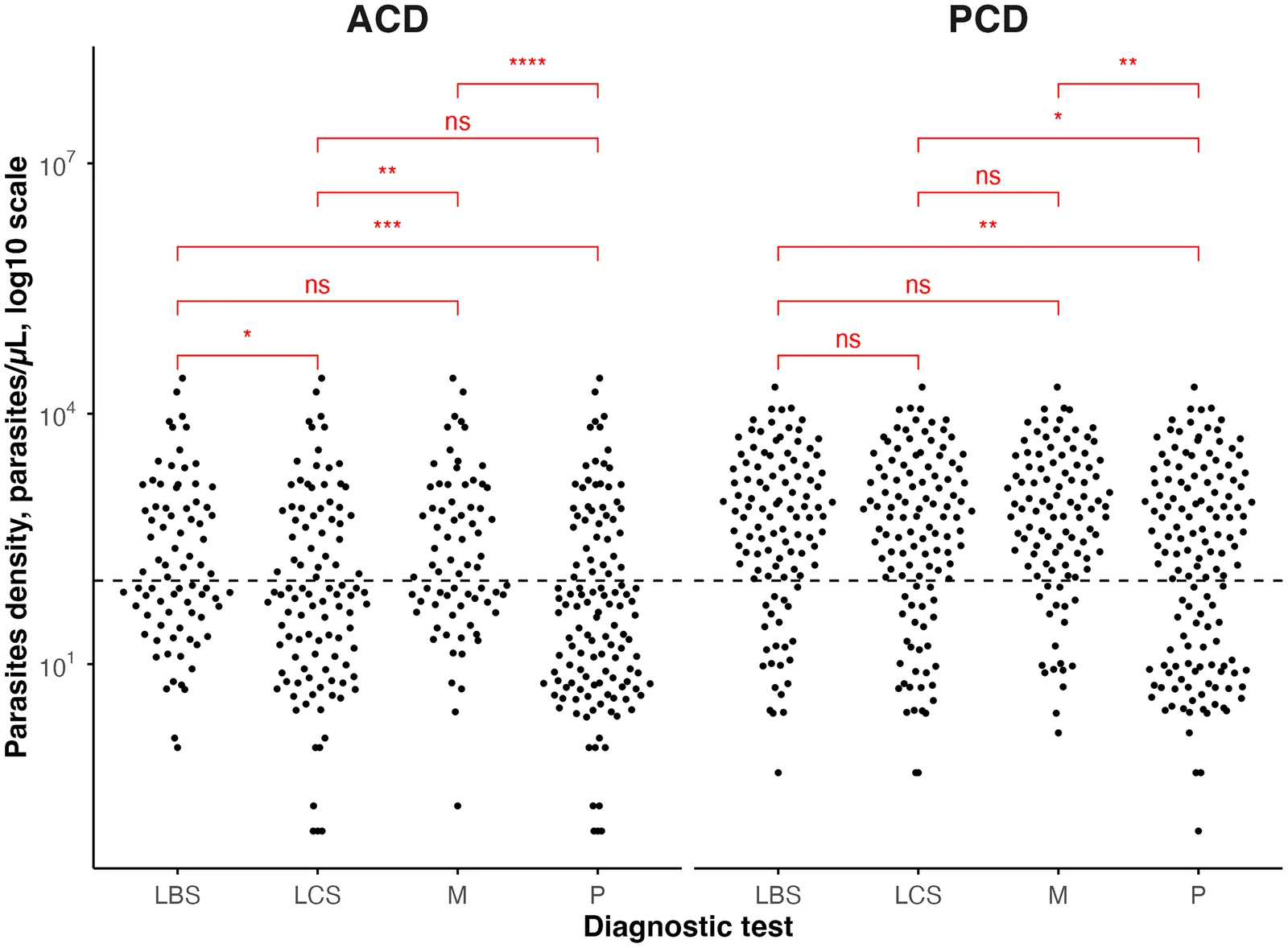
Malaria parasite density by diagnostic test. Each point represents the qPCR parasitemia of samples positive by the respective diagnostic test (LBS, LCS, M, and **P)**. The dotted line indicates the 100 parasites/uL limit. Abbreviations: M, microscopy; LBS, cLAMP using BS DNA; LCS, cLAMP using MSC DNA; P, polymerase chain reaction; ACD, active case detection; PCD, passive case detection.

We performed statistical comparisons using the Wilcoxon rank-sum test with Bonferroni corrections within each collection group (ACD and PCD). * = p < 0.05; ** = p < 0.01; *** = p < 0.001; **** = p < 0.0001; ns = p > 0.05.

## Discussion

The persistence of malaria transmission in hard-to-reach regions of the Peruvian Amazon continues to pose substantial challenges to achieving elimination [1,2]. Limited access to healthcare services, combined with operational constraints in the field, restricts the effectiveness of routine surveillance. Moreover, previous studies have shown that microscopy and RDTs have limited sensitivity for detecting low-density infections, which are increasingly prevalent as transmission declines [9–11,28,29]. These diagnostic gaps likely contribute to underestimating the malaria burden and ongoing transmission, underscoring the importance of strengthening diagnostic performance in the field to improve case detection and support malaria elimination efforts.

In this study, we evaluated a cLAMP assay based on a neutral red color change using DBS samples collected via both ACD and PCD in remote, rural, malaria-endemic communities. Notably, this evaluation was conducted under laboratory conditions with DBS samples transported from the field; therefore, it should not be interpreted as a point-of-care implementation. By analyzing both strategies, we assessed the assay’s capacity to uncover hidden infectious cases by detecting asymptomatic cases, while validating the assay on samples from symptomatic patients seeking care in a remote community.

The use of neutral red allowed clear visual discrimination between positive and negative reactions, reducing ambiguity associated with other colorimetric indicators such as metal-ion–sensitive dyes or malachite green, which can exhibit variable color intensity and complicate result interpretation under field conditions [15,30,31]. The consistent color change from yellow to fuchsia provides a stable and easily interpretable signal, an advantage in field settings, where lighting conditions, operator experience, and workload may influence the interpretation of results [22].

Our results demonstrate that the diagnostic performance of cLAMP is strongly influenced by the DNA extraction method, a factor known to affect analytical sensitivity in isothermal amplification assays [22,32]. cLAMP using MSC DNA achieved higher sensitivity and diagnostic accuracy than cLAMP using BS DNA and microscopy across both PCD and ACD, particularly among samples with parasite densities below 100 parasites/µL. This difference likely reflects improved inhibitor removal and more efficient nucleic acid recovery with column-based extraction methods, which are critical for reliable amplification in low-parasitemia infections [32]. In contrast, microscopy exhibited the lowest sensitivity and overall accuracy in low-parasitemia infections, underscoring its well-known limitations in detecting asymptomatic and submicroscopic infections commonly identified during active surveillance [28,29]. These findings align with previous reports documenting reduced microscopy sensitivity under low-transmission conditions and highlight the critical need for sensitive molecular diagnostics to accurately identify low-density reservoirs of infection and sustain malaria elimination efforts [28,29].

Across both ACD and PCD strategies, cLAMP using MSC DNA demonstrated the highest diagnostic accuracy relative to qPCR, with an AUC value of 0.91. In contrast, cLAMP using BS DNA showed moderate performance, with AUC values ranging from 0.82 to 0.88 across surveillance contexts. The reduced sensitivity observed with BS-derived DNA was primarily attributable to decreased DNA yield and incomplete removal of amplification inhibitors inherent to crude extraction methods, particularly affecting samples with parasite densities below 100 parasites/µL as quantified by qPCR. Previous evaluations of cLAMP and related LAMP assays using whole blood samples have reported similar reductions in sensitivity with simplified extraction approaches, underscoring the importance of DNA quality and extraction efficiency for reliable detection of low-density infections [22,32].

The diagnostic performance of the cLAMP assay depends on the surveillance strategy and extraction method. In ACD samples, where qPCR confirmed a higher prevalence of low-parasitemia infections, cLAMP using MSC DNA significantly outperformed microscopy. These findings likely reflect the combined effects of low parasite density and increased diagnostic workload during ACD, factors known to reduce microscopy sensitivity due to reader fatigue and operator-dependent variability [1,28,29]. In contrast, PCD samples typically exhibited higher parasite densities and lower daily testing volumes, resulting in similar performance between microscopy and cLAMP, regardless of extraction method. Our findings suggest that while microscopy remains adequate for routine clinical diagnosis, cLAMP can complement conventional microscopy and offers a more robust approach when combined with column-based DNA extraction during ACD screenings, where subclinical infections are the primary challenge.

The diagnostic accuracy of our cLAMP methods was partially consistent with previous LAMP-based evaluations in the Peruvian Amazon. Most Peruvian studies evaluated their tools on PCD samples, typically from symptomatic infections with higher parasitemias. For instance, Barazorda et al. tested a malachite green LAMP (MG-LAMP) assay on PCD samples using BS extraction and reported notably higher sensitivity than ours (sensitivity: 99.4% [95.5%; 100%]; specificity: 97.1% [91.9%; 99.4%]); however, this may reflect the use of expert microscopy as the gold standard rather than PCR [22]. Similarly, another study by the same group, using the MG-LAMP assay with column-based extraction on PCD samples, reported sensitivity comparable to our cLAMP (sensitivity: 92% [85%; 96.5%]; specificity: 100% [89.1%; 100%]), highlighting the importance of high-quality DNA extraction methods [20].

Compared with PCD, ACD enables us to detect asymptomatic individuals who are more likely to harbor low-density submicroscopic infections. To date, two studies have explored the diagnostic performance of LAMP-based assays on ACD samples in the Peruvian Amazon. In our previous work, we studied the diagnostic performance of our cLAMP assay using whole blood samples and found an even lower sensitivity (sensitivity: 47.3% [38.77%; 55.9%]; specificity: 100% [100%; 100%]) than in the present work using DBS, likely due to context-specific conditions [23]. Another study using the LoopAmp detection kit in ACD samples found a higher sensitivity than our method (sensitivity: 91.8% [87.7%; 94.9%]; specificity: 91.9% [87.8%, 95.0%]) despite using a similar extraction method, probably because of using whole blood samples, which also leads to several logistic challenges rendering implementation in hard-to-reach communities like Nueva Jerusalén unfeasible [19]. Implementation assays of the LoopAmp kit showed a significant reduction in diagnostic performance in riverine rural communities compared to those accessible by road, due to limited accessibility in this setting and the logistical limitations of using whole blood samples [19]. In such settings, the use of whole blood critically limits the implementation of LAMP assays, and more practical samples, such as DBS, could overcome this problem.

The strength of our cLAMP lies in using DBS samples with column-based DNA extraction, allowing us to outperform microscopy in ACD activities while overcoming the limitations of whole blood, reinforcing its value in elimination contexts. Furthermore, the colorimetric identification of *Plasmodium spp.* infections eases the interpretation of results by trained health workers. Previous studies were limited by the use of fluorescent approaches, which can render unclear results and compromise accuracy. Notably, including a DNA concentration step, as conducted by Barazorda et al., could improve the performance of BS DNA extraction and further increase the sensitivity of our assay. From a cost perspective, LAMP assays offer several advantages over qPCR. Although qPCR remains the most analytically sensitive method, it requires specialized thermocyclers, stable electricity supply, advanced laboratory infrastructure, and highly trained personnel, all of which substantially increase implementation costs in remote settings [32,33]. In contrast, LAMP assays can be performed using portable heating devices, operate under isothermal conditions, and allow visual detection without expensive fluorescence readers, reducing equipment and maintenance costs [32,34]. Still, LAMP assays require several laboratory steps that limit their immediate use as a stand-alone diagnostic test, highlighting their role as a complementary molecular tool for active surveillance and elimination strategies instead of a replacement for microscopy.

While column-based DNA extraction entails greater per-sample costs than boil-and-spin protocols, its enhanced sensitivity during active surveillance may justify the additional expenditure by improving case detection and limiting the persistence of low-level transmission. Overall, our cLAMP assay represents an intermediate diagnostic option between microscopy and qPCR, balancing sensitivity and field adaptability for use in ACD, increasing the number of additional true cases found compared with microscopy. Despite the lack of a formal cost-effectiveness analysis, we expect our tool to follow the same trend as other LAMP-based assays.

As other studies state, the advantage of LAMP-based tools does not rely solely on their lower costs and higher sensitivity than microscopy and RDT, but also on their potential to increase the sample processing capacity [18]. LAMP-based assays have been shown to reduce the turnaround time and improve the processing capacity at health facilities in rural communities. While microscopy is limited by the microscopist’s skill and fatigue, LAMP assays require minimal molecular laboratory training and can be scaled by hiring additional personnel and acquiring additional equipment [22]. In our study, we further extend the adaptability of our cLAMP by demonstrating higher sensitivity than microscopy in ACD using MSC DNA from DBS samples, thereby reducing limitations related to cold-chain storage, transport logistics, and biosafety risks during whole-blood sample handling and shipment. Notably, this study did not evaluate RDT diagnostic performance; therefore, our comparisons are limited to microscopy, cLAMP, and qPCR. Nonetheless, previous research shows that RDT sensitivity declines as the parasite density decreases, limiting the detection of low-density infections [9–11].

Several limitations of our cLAMP assay should be acknowledged. First, the cLAMP assay provides a qualitative result based on visual color change and does not quantify parasites, limiting its use in settings requiring quantitative assessment of parasitemia. Second, as with other LAMP assays, we observed false-positive results: three samples were qPCR-negative but cLAMP-positive. False positives could arise from stochastic distribution of target molecules between aliquots in samples near the limit of detection, increased detection probability due to multicopy targets, nonspecific amplification, primer interactions, or carry-over contamination. Third, although DBS facilitates sample collection, storage, and transport from rural settings, the current cLAMP workflow still includes DNA extraction, incubation, molecular training, and quality control procedures, limiting its use as a standalone point-of-care diagnostic. Fourth, the cLAMP assay did not incorporate an internal amplification control (IAC), which limits the ability to distinguish true negatives from false negatives caused by amplification inhibition or assay failure. This limitation is particularly relevant for low-parasitemia samples, in which the target DNA concentration may approach the detection limit of the assay and the effect of inhibition or inefficient amplification may be more consequential. Consequently, some negative cLAMP results cannot be independently confirmed as true negatives based on amplification performance, and the possibility of false-negative results cannot be completely excluded. Incorporating an appropriate IAC in future cLAMP evaluations would provide a means of monitoring amplification integrity and improve confidence in negative results, thereby allowing a more robust assessment of assay sensitivity, particularly for low-density infections.

Collectively, these results underscore the importance of selecting diagnostic tools based on the surveillance context and parasite burden. Because active surveillance often detects low-density and asymptomatic infections that contribute to residual transmission [28,29], false negatives can lead to missed infections and an underestimation of the malaria burden. Undetected infections may remain untreated or insufficiently monitored, allowing residual transmission to persist within communities. Therefore, optimizing molecular approaches such as cLAMP, in combination with efficient DNA extraction, may improve detection of low-density infections that could otherwise be missed, strengthen surveillance, and support interventions aimed at interrupting residual transmission and advancing malaria elimination. Future studies should explore the field implementation of our assay within ACD activities conducted in collaboration with the MoH, assess its cost-effectiveness and turnaround times, and optimize simplified DNA extraction methods that balance sensitivity with field applicability. By improving diagnostic sensitivity and accessibility while serving as a complementary tool to microscopy, cLAMP has the potential to contribute meaningfully to sustained malaria control and elimination efforts in Peru and similar endemic settings.

## Conclusions

In conclusion, this study demonstrates that cLAMP is a valuable complementary diagnostic tool for malaria detection in endemic and remote settings. cLAMP using BS DNA showed diagnostic performance comparable to microscopy and may serve as a complementary tool in contexts with limited laboratory infrastructure and a shortage of skilled personnel. In contrast, cLAMP using MSC DNA achieved significantly higher sensitivity than microscopy, although it did not reach the analytical sensitivity of qPCR.

The high proportion of low-parasitemia infections identified during ACD underscores the limitations of routine microscopy and highlights the need for more sensitive diagnostics. In this context, integrating cLAMP into active surveillance could reduce missed infections, strengthen malaria detection, shorten the transmission period, and improve case prognosis, particularly in elimination settings. The use of DBS further enhances the operational feasibility of cLAMP by simplifying sample collection, storage, and transportation in hard-to-reach areas.

## Supporting information

S1 File Dataset with sample codes, community, collection type, microscopy, PCR and cLAMP results. (XLSX)

S2 File Map of cLAMP primer locations on an alignment of reported sequences. (PDF)

S3 File Table including the cLAMP primer sequences and targets. (DOCX)

## Abbreviations

COX1: Cytochrome oxidase I
DNA: Deoxyribonucleic acid
EDTA: Ethylene diamine tetra-acetic acid
HRP2: Histidine-rich protein 2
LAMP: Loop-mediated isothermal amplification
LOD: limit of detection
MG-LAMP: Malachite green colorimetric LAMP
PCR: Polymerase chain reaction
PoC: Point of care
SDS: Sodium dodecyl sulfate
TEN buffer: Tris–EDTA Sodium chloride buffer
