# Supplementary material for: Detection of Plasmodium vivax and Plasmodium falciparum using colorimetric loop-mediated isothermal amplification from dried blood spots collected in active and passive malaria surveillance in the Peruvian Amazon
Source: PLoS Negl Trop Dis. 2026 Oct 13;20(10):e0014366. doi: 10.1371/journal.pntd.0014366 (PMC13712756; doi:10.1371/journal.pntd.0014366)
Supplement: Supplementary file 1 [file pntd.0014366.s002.pdf]

Alignment of *Plasmodium vivax COX1* sequences, showing the primer recognition regions and the most common variations found in the sequences

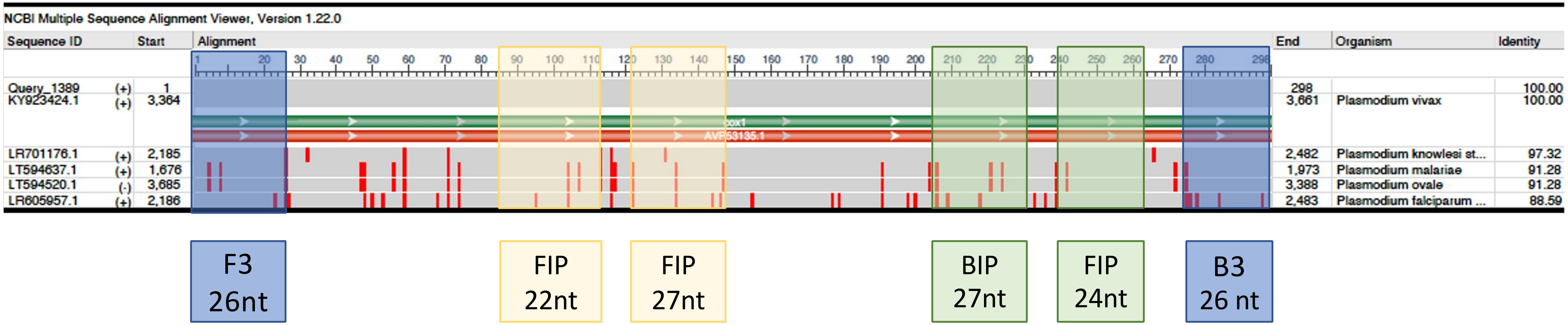

F3 1-26	26
B3 273-298	26
FIP 84-105	22
121-147	27
BIP 204-230	27
240-263	24

**COX1**	**F3**	**GAATAATTGCACAAGAAAATGTTAAC**
	B3	GCAACAGGAGATAAAGACATAAGTGA
	FIP	CAAGTTCTGGAGAACCACATAAAATTG–CCAGGATTATTTGGAGGATTCG
	BIP	GTCATTTTATCTACAGCAGCAGAATTT-TGGTGGATATAAAGTCCATCCAGT

Alignment of *Plasmodium falciparum Pfr364* sequences, showing primer recognition regions and the most common variations found in the sequences

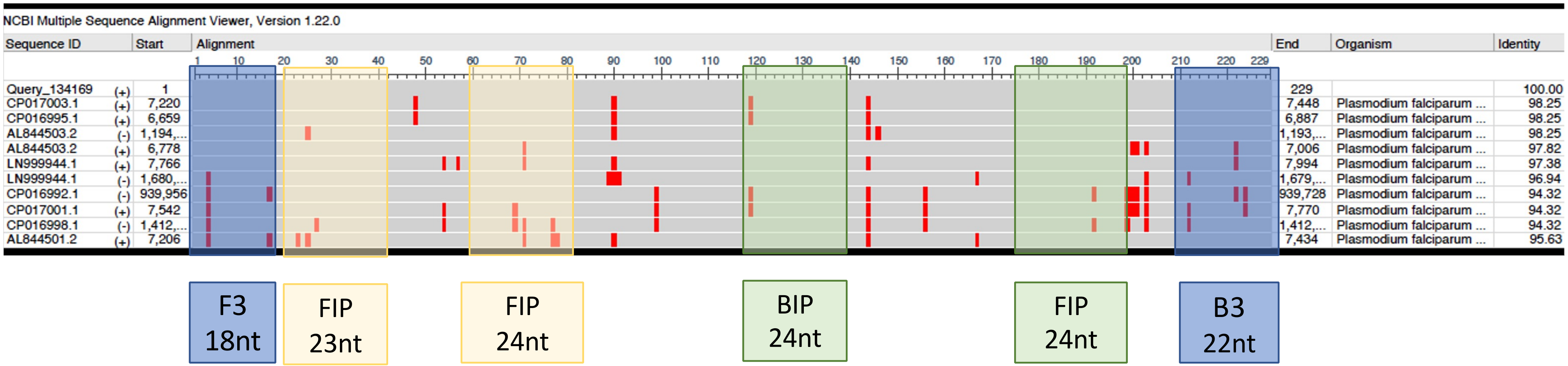

F3 1-18	18
B3 208-229	22
FIP 59-82	24
19-41	23
BIP 117-139	24
176-199	24

**Pfr364**	**F3**	**CACTAGGTACGCCAACAT**
	B3	ACCCACAATTTTGATTGAGATG
	FIP	GTAGACACCATATGGTACCACGTA-GGATGTGTCTATCATATAGTCCG
	BIP	TTGTACCCAATTTTCCCCTAGCA-GTGTGTGCAACATCATAATCATCA
