# Supplementary material for: Detection of Plasmodium vivax and Plasmodium falciparum using colorimetric loop-mediated isothermal amplification from dried blood spots collected in active and passive malaria surveillance in the Peruvian Amazon
Source: PLoS Negl Trop Dis. 2026 Oct 13;20(10):e0014366. doi: 10.1371/journal.pntd.0014366 (PMC13712756; doi:10.1371/journal.pntd.0014366)
Supplement: Supplementary file 2 [file pntd.0014366.s003.docx]

| **Species** | **Assay** | **Target** | **Primer** | **Sequence (5′–3′)** |
| --- | --- | --- | --- | --- |
| *P. vivax* | PvCOX1-LAMP | COX1 | F3 | GAATAATTGCACAAGAAAATGTTAAC |
|  |  |  | B3 | GCAACAGGAGATAAAGACATAAGTGA |
|  |  |  | FIP | CAAGTTCTGGAGAACCACATAAAATTG–CCAGGATTATTTGGAGGATTCG |
|  |  |  | BIP | GTCATTTTATCTACAGCAGCAGAATTT–TGGTGGATATAAAGTCCATCCAGT |
| *P. falciparum* | Pfr364-LAMP | Pfr364 | F3 | CACTAGGTACGCCAACAT |
|  |  |  | B3 | ACCCACAATTTTGATTGAGATG |
|  |  |  | FIP | GTAGACACCATATGGTACCACGTA–GGATGTGTCTATCATATAGTCCG |
|  |  |  | BIP | TTGTACCCAATTTTCCCCTAGCA–GTGTGTGCAACATCATAATCATCA |
